# A large randomized trial assessing alcohol cessation versus moderation on clinical events: UNATI

**DOI:** 10.1007/s10654-025-01330-w

**Published:** 2026-07-11

**Authors:** Miguel Ángel Martínez-González, Principal Investigators of the UNATI trial, María Barbería-Latasa, Zenaida Vázquez-Ruiz, Miguel Angel Alvarez-Mon, Diego Martínez-Urbistondo, Miguel Ruiz-Canela, Maira Bes-Rastrollo, Miguel A. Martínez-González, Jesús Antonio Vaquero-Cruzado, Javier Quintero, Albert Bellvert, Maria Teresa Barrio-López, Marc Vives, Juan Pablo Chart, Aitor Hernández-Hernández, Cristina Carretero, Juan Manuel Zubiría, Nieves Lopez-Laguna, Victor Vallejo-García, F. Javier Basterra-Gortari, Alejandro Fernandez-Montero, Victoria Güeto, Daniel Cabo-Navarro, Manuel Landecho, Vicente Martín-Sanchez, Francisco Fanjul, Juan Diego Sánchez-Vega, Sonia Eguaras, David Gurpegui, José María Mora, Ma. Isabel Sonia Martín-Almendros, Nuria Villamor Sagredo, Nuria Alonso-Santander, R. Valenti-Azcárate, Julio Herrero-Colomin, José Javier Varo, Adela Navarro, Pablo Bazal-Chacon, Ana García-Arellano, Mercè Lopez, Barbara Vizmanos, Guillermo Lahera, Rosa M. Molina-Ruiz, A. Huerta-Gonzalez, A. Bajo-Buenestado, E. Molano-Alvarado, Fernando Mora, María Llavero-Valero, Eva M°. Raidó Quintana, Josep Montserrat-Capdevila, Eileen Cordoves Maurisset, Alberto Esteban-Fernández, Iñigo Rubio, Borja Domingo-Cardenal, Manuel García de Yébenes, Julia Navarro-Fernández, Esther Gomez-Cordero

**Affiliations:** 1 Centro de Investigación Biomédica en Red de Fisiopatología de la Obesidad y Nutrición (CIBEROBN), Instituto de Salud Carlos III (ISCIII), Madrid, Spain; 2 Department of Preventive Medicine and Public Health, School of Medicine, University of Navarra, Pamplona, Spain; 3 Navarra Institute for Health Research (IdiSNA), Pamplona, Spain

**Keywords:** Moderate alcohol consumption, Randomized controlled trial, Mediterranean drinking pattern, Alcohol cessation, UNATI study

## Abstract

The health effects of moderate alcohol consumption remain a highly debated topic in public health and epidemiology. While observational studies have suggested a possible protective effect of moderate drinking, particularly within Mediterranean patterns, concerns about confounding and selection bias persist. No randomized trial to date has definitively addressed whether alcohol cessation offers superior health benefits compared to sustained moderate drinking. In response to this evidence gap, the European Research Council has funded the University of Navarra Alumni Trialist Initiative (UNATI), a large, pragmatic, randomized controlled trial designed to assess the long-term effects of two behavioral interventions: complete alcohol cessation versus moderated consumption following a Mediterranean pattern. UNATI will enroll over 10,000 adults in Spain (men aged 50–70 and women aged 55–75) who currently consume alcohol regularly. Participants will be randomly assigned to one of the two interventions for four years, with annual clinical evaluations and lifestyle assessments. The trial’s hybrid design—combining centralized coordination with a network of regional medical professionals—offers a unique opportunity to generate high-quality causal evidence in a real-world setting. If successful, UNATI could become a landmark study, helping to resolve a decades-long controversy regarding moderate alcohol use and health.

## Scientific basis for a big question: abstinence or moderation?

The relationship between alcohol consumption and health has been debated for years, but it became especially controversial after the 2018 report from the Global Burden of Disease (GBD) [1, 2] which concluded that the level of ethanol consumption that minimizes harm is always zero. However, in their subsequent 2022 updated report [3], the same group introduced region-, sex-, and age-specific estimates, and concluded that, in older adults –given their underlying higher cardiovascular risk—light-to-moderate alcohol consumption could offer net benefits, highlighting the need for more nuanced recommendations instead of universal uniform guidelines. This controversy deepened in 2025 when almost simultaneously a Surgeon General Advisory report [4] warned about the link between even low levels of consumption and the risk of seven types of cancer, and another report from the National Academies [5] acknowledged both potential cardiovascular benefits and health risks, including a statement that moderate alcohol drinking is associated with reduced all-cause mortality. On the other hand, Mendelian randomization (MR) studies [6, 7] have reported no health benefits of moderate alcohol intake, calling into question previous conventional epidemiological findings, always under a number of assumptions that can be doubtful [8–10], and which do not allow to assess the causal effect of the overall drinking pattern [11].

In light of these controversial findings, there is an urgent need for large-scale randomized clinical trials in adult drinkers, comparing cessation with moderation. While small trials have shown some metabolic and cardiovascular benefits of moderate red wine consumption, a large-scale trial comprehensively assessing hard clinical outcomes has not yet been conducted. A balanced, evidence-based approach—free from bias, independent of industry, and grounded in rigorous research—is essential for informed public health policy decisions.

Fortunately, in 2023, the European Research Council funded the University of Navarra Alumni Trialist Initiative (UNATI), a large randomized clinical trial aimed at evaluating the health effects of alcohol cessation (versus moderation), through a 5-year Advanced Research Grant (December 2023–November 2028). The design is a 4-year non-inferiority trial that will randomly assign more than 10,000 alcohol-consuming adults (50–75 years) living in Spain.

## A hybrid recruitment model

**Fig. 1:**
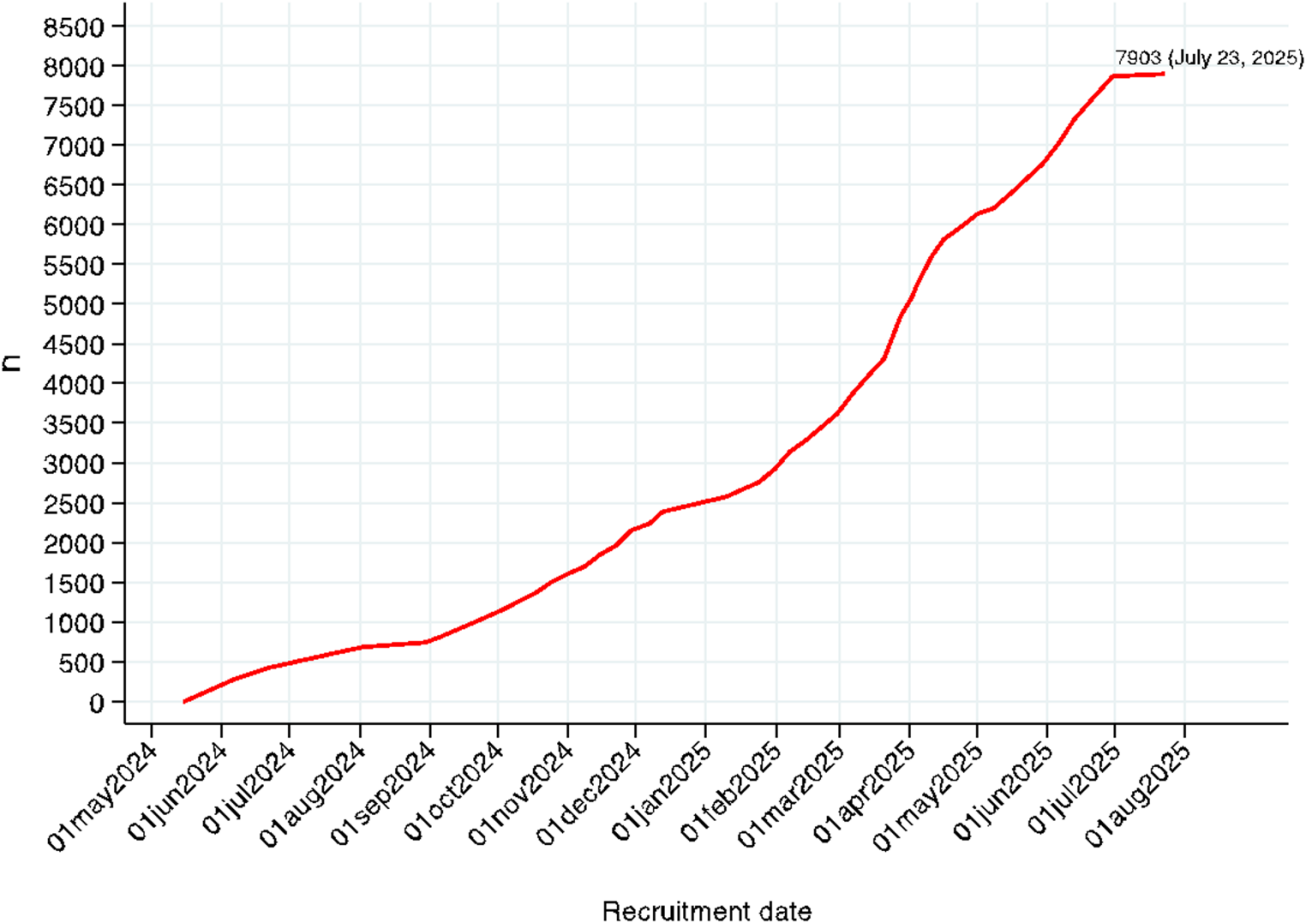
Recruitment dates for the first 7903 participants in the UNATI trial

In this article, we summarize the current status of the UNATI trial. The trial was registered on ClinicalTrials.gov (NCT06338215) on March 2024. Between May 2024 and July 23, 2025, 7903 participants were recruited (Fig. 1).

Initially, our main plan was to recruit participants through physicians’ clinics, but due to initial slow enrollment, we decided to turn to the media to promote the trial. Major Spanish newspapers, some television channels, social media and radios provided wide coverage, including weekend newspaper features up to two pages long. On social media, we managed to get influencers with hundreds of thousands—and in some cases, millions—of followers to help spread the word. Thanks to these efforts, a large number of potential candidates expressed interest in the project and contacted us to participate. They were referred to the approximately 500 medical doctors involved in the project as “trialists”. Each medical doctor contacted potential candidates recruited through the media to assess their eligibility to enter the trial. Among the 7903 participants who met the inclusion criteria and were recruited, approximately 2000 had only answered the eligibility questionnaire and had have the first contact with the medical doctor as of July 2025, but they were in the waiting list to have the first session with the coach (see below) and to be randomized.

Participants should meet the following inclusion criteria: current drinkers of any alcoholic beverage consuming between ≥ 3 and ≤ 40 drinks per week (approximately 30 to 400 g of pure alcohol per week), aged 50–70 years (men) or 55–75 years (women), non-institutionalized, with a life expectancy of over 5 years (as judged by their physicians), and willing to receive 4 years of guidance on making their alcohol consumption healthier. They also needed to be familiar with information technologies. Candidates with prevalent cardiovascular disease, diabetes, cancer, or a history of depression were eligible. Exclusion criteria included severe psychiatric conditions, a medical diagnosis of liver cirrhosis, cognitive impairment/dementia, use of medications incompatible with any alcohol intake, or a history (within the past 10 years) of liver or breast cancer. No participant will be encouraged to start or increase their alcohol consumption.

Participants must own a smartphone and a computer (or tablet) with internet access and be highly familiar with videoconferencing tools. A particularly innovative feature of this trial was that the interventions were conducted remotely. We gained extensive experience with this methodology for nutritional and lifestyle interventions during the successful implementation of the remote PREDIMAR trial [12] and its use in the PREDIMED-Plus trial [13, 14] during the pandemic lockdown—both of which achieved major changes in overall dietary patterns through behavioral intervention.

The *primary aim of UNATI* is to assess, in current drinkers (men aged 50–70 or women aged 55–75 who consume ≥ 3 but ≤ 40 drinks/week), the non-inferiority of a 4-year harm-reduction intervention (moderation) compared to an intervention aimed at promoting abstinence. Both interventions include quarterly follow-up remote contacts with coaches to support either moderation or abstinence. In the case of moderation, participants are guided to follow a Mediterranean Alcohol Drinking Pattern (MADP) [15], which involves moderate intake only (≤ 7 drinks/week for women and ≤ 14 drinks/week for men), avoiding binge drinking, with a preference for red wine, always consumed with meals and spread throughout the week. The active comparator arm is a 4-year intervention program promoting reduction in all types of alcoholic beverages with the aim to attain complete abstinence, also delivered through quarterly contacts.

The *primary outcome* is a composite endpoint that includes: all-cause mortality, cardiovascular events, any invasive cancer, liver cirrhosis, type 2 diabetes, depression, dementia, injuries requiring hospitalization, and infections requiring hospitalization. As a secondary analysis, more severe outcomes (mortality, invasive cancer, stroke, myocardial infarction, liver cirrhosis) will be analyzed independently with sufficient priority over less severe outcomes using the win-ratio method [16].

Beyond the primary objective, the UNATI trial includes several scientifically and practically relevant *secondary objectives*. First, it aims to establish a national network of approximately 500 physicians (trialists), mostly medical doctors, alumni of the University of Navarra, to facilitate the recruitment of participants for randomized trials focused on lifestyle prevention strategies. This collaborative model offers an innovative platform for large-scale clinical research. So far, participating physicians have shown great interest, partly due to their sense of belonging to their alma mater and the opportunity to reconnect with former classmates and professors.

From a *clinical perspective*, the study will test the non-inferiority of moderation versus abstinence in a variety of intermediate health markers, including anthropometric parameters, liver enzymes, blood pressure, lipid and glucose profiles, depressive symptoms, problematic alcohol use, cognitive function, and quality of life. It will also independently assess major clinical outcomes such as all-cause mortality, type 2 diabetes, cardiovascular events, serious injuries, and depression. Another key goal is to monitor the 10-year incidence of invasive cancers (excluding non-melanoma skin cancer), thus providing valuable long-term insights.

From a *patient-centered perspective*, the UNATI trial will evaluate the actual adherence achieved in each trial arm, the impact of observed adherence on primary outcomes, and the predictors of compliance with either intervention, analyzed separately for men and women. This approach is intended to foster more personalized and realistic public health recommendations based on actual participant behavior.

## Trialists, coaches, and organizational structure

To carry out this project, we have established a network of 36 physicians referred to as *Coordinators*. These Coordinators are responsible for recruiting participants, conducting the annual review of medical records, and collecting clinical information on events and other parameters during follow-up. Each coordinator, in turn, has formed a group of between 7 and 28 physicians—referred to as *trialists*—who assist them in meeting recruitment targets. Several of the coordinators had previously participated in major lifestyle trials such as PREDIMED [17, 18], PREDIMED-Plus [13], and PREDIMAR [12].

Using funds from the European Research Council grant, we have hired a team of 15 *coaches* (psychologists, nutritionists, one nurse, and one physician) to deliver the remote quarterly interventions. Many of the coaches already had experience in similar trials, but all received specific training before the recruitment began. In addition, they participate in weekly continuing education sessions, which also serve as a space to exchange ideas, impressions, and experiences.

We have extensive experience in training, certifying, and supervising healthcare professionals to conduct complex behavioral interventions aimed at producing substantial dietary and beverage intake changes among older adults [19–21]. Particular attention has been paid to ensuring that all coaches are capable of delivering both intervention arms with equal quality, and that their personal views regarding alcohol consumption (e.g., whether favoring abstinence or moderate red wine intake) do not influence the standardized protocol. All coaches have been trained to implement both interventions faithfully, regardless of personal beliefs.

*In the abstinence arm*, participants are receiving four yearly contacts (group and individual teleconferences lasting 30–45 min) with the eventual goal of completely quitting alcohol. A reduction of at least 60% from baseline consumption is considered sufficient, and alcohol-free days will be monitored. If no improvement is observed after 1 year in any participant randomized to this arm, a contingency personal plan will be implemented. *In the moderation arm*, the contacts will be shorter (15–20 min) and will focus on promoting a Mediterranean drinking pattern: not exceeding 7 drinks per week for women or 14 for men, spreading out consumption, drinking only red wine, doing so with meals, and avoiding binge drinking [15, 22]. Increasing alcohol intake will never be encouraged.

The trial is overseen by a five-member *Steering Committee*, composed of the Principal Investigator, two full professors from the Department of Preventive Medicine at the University of Navarra, and two practicing physicians who are also engaged in clinical research. An independent *Data and Safety Monitoring Board* (*DSMB*), composed of five well-known and highly respected academics, monitors the study’s progress and ensures compliance with safety and ethical standards. In addition, a group of 17 *external advisors* are met individually with the team at least once a year to provide input and scientific guidance.

## Assessments and measurements

Trialists (medical doctor) conduct the first contact and interview eligible candidates to inform them about the study and complete a brief screening questionnaire. If inclusion criteria are met, participants are invited to sign the informed consent form by the coaches. Once informed consent is obtained, at which point randomization takes place, the coach deliver the first interview. During this initial interview, participants complete a long battery of questionnaires, assisted by the coach, and they are informed of their assigned group. They also receive initial instructions and motivational counseling. In this first contact the persuasion in the abstention group is mainly focused on reducing intake rather than attaining immediate cessation. The emphasis on total cessation is reserved for the intervention carried out at six months.

At baseline participants—assisted by the coach—complete questionnaires including sociodemographic information, lifestyle habits, medical history, medication use, and a validated 143-item food frequency questionnaire FFQ [23]. In addition, they complete on their own (online, at home) the following instruments: a validated physical activity questionnaire [24], a sleep habits questionnaire (including the Epworth Sleepiness Scale), the Beck Depression Inventory-II, a quality of life questionnaire (SF-36), and the Human Flourishing questionnaire developed by Vanderweele [25].

During the baseline visit, the trialist (i.e., the participant’s physician) records anthropometric data, systolic and diastolic blood pressure, and conducts several tests, including an electrocardiogram (ECG), cell blood count, fasting blood lipid profile, liver enzymes, and fasting glucose. Additionally, trialists conduct an annual review of medical records to identify hospital admissions and the diagnosis of any significant clinical events specified in the protocol. The assessments and questionnaires are repeatedly collected during the 4 year duration on the trial, on a yearly basis.

In-person follow-up visits with the trialist are scheduled at baseline and at years 1, 2, 3, and 4 (end of intervention). All questionnaires are also available online, allowing participants the option to complete them electronically at any point. Furthermore, we are collecting hair samples and urine samples to measure ethyl glucuronide (EtG) in a subsample of 600 participants, providing an objective repeated biomarker of alcohol intake. The resulting dataset (fully anonymized) will be made available to any qualified researcher who submits a relevant proposal to the Steering Committee for secondary data analysis. All standard legal safeguards for the protection of individual data will be observed.

## Current challenges

*First, to reach our target of 10,000 participants* recruitment will be resumed in January 2026. *Second, as in any longitudinal study, losses to follow-up may pose a threat to validity*. Our plan to maximize retention is based on the commitment of highly selected trialists and coaches and to foster a sense of community and involvement among the selected participants, through the periodic use of newsletters. A potential limitation is the possibility that participants may not accurately report their alcohol consumption or may tend to underreport it during conversations with coaches or in self-administered questionnaires due to social desirability bias. To mitigate this risk, we will measure objective biomarkers of alcohol intake (HDL cholesterol) in all participants, and hair ethyl glucuronide in a subsample. In addition, both the abstinence and moderation arms will be informed that objective biomarkers will be used to verify self-reported alcohol intake.

*It is possible that the trial may not last long enough to detect effects on certain outcomes, such as cancer incidence*. We acknowledge this possibility and assume that extended follow-up of up to 10 years may be needed, for which additional funding will be sought. However, for most outcomes, four years of follow-up will be sufficient, as the trial by Voskoboinik et al. [26] on atrial fibrillation observed effects after only six months.

Finally, it is crucial to attain high adherence to the intervention so as the contrast in mean alcohol intake between groups may be sufficiently high, at least 9–10 g/d of difference between the two arms after one-year follow-up. This is needed for the validity of the trial, particularly given its non-inferiority design. For this reason, we will include a per-protocol analysis. More importantly, we have invested heavily in the meticulous training and motivation of the coaches to deliver a highly persuasive, high-quality behavioral intervention, particularly after the first 6 months, once the participant is acquainted with the coach. This persuasive advice will be sustained over the full four years. Our target of achieving an abstinence rate of at least 50% in the abstinence arm (or a 60% reduction in mean alcohol intake) appears feasible and will provide sufficient contrast for the intention-to-treat approach, which will be the primary analysis.

As briefly mentioned in several leading journals [11, 27, 28], the UNATI randomized trial will provide, for the first time, high-level evidence to answer the important pragmatic question of what is the healthiest option when advising drinkers aged 50–75 years in clinical settings regarding alcohol intake. This trial will also offer valuable insights into how to deliver practical advice for changing drinking behaviors. The broad behavioral approach adopted in this trial aligns with the most promising path to reversing the alarming decline in public health gains over recent decades, as wisely suggested by Narayan et al. [29].
